# RISK FACTORS AND PROTECTIVE FACTORS OF VICTIMS OF ELDER ABUSE IN THE PHILIPPINES

**DOI:** 10.1093/geroni/igae098.0955

**Published:** 2024-12-31

**Authors:** Victoria Apuan, Carmelina Oyales

**Affiliations:** Miriam College, Quezon City, Rizal, Philippines; Miriam College and Eunoia Educational Consulting, Quezon City, National Capital Region, Philippines

## Abstract

In a country where the prevailing culture puts a premium on respect and care for the elderly, elder abuse is an interesting phenomenon. The study focuses on elder abuse and the different forms of abuse occurring in Filipino communities. Following recommendations of previous studies, the authors undertook a qualitative study composed of individual interviews of ten victims of elderly abuse and focus group discussions with thirty two local government officials. Ageism and lack of awareness of the human rights of elders were identified as deterrents to the prevention of abuse of elders within families and blocked their access to socio-psychological and economic help. Aside from ageism and the social isolation of the victims of elder abuse, risk factors also included material poverty, problematic family dynamics such as children’s lack of attachment to parents, lack of support of extended family members, and the lack of awareness that the elderly have human rights. Protective factors needing to be established and strengthened were community-based monitoring and reporting system of elder abuse, a current survey of household with senior citizens, more stringent enforcement of existing laws protecting the elderly human rights education in schools, more frequent visits of social workers to communities, especially those in the hard to reach areas. Keywords: elder abuse, aging population, ageism, poverty, senior citizens human rights.

